# Supplementary material for: The genome sequence of the scarce swallowtail, Iphiclides podalirius
Source: G3 (Bethesda). 2022 Aug 5;12(9):jkac193. doi: 10.1093/g3journal/jkac193 (PMC9434224; doi:10.1093/g3journal/jkac193)
Supplement: jkac193_Supplementary_Material [file jkac193_supplementary_material.pdf]

386 8 Supplementary Materials

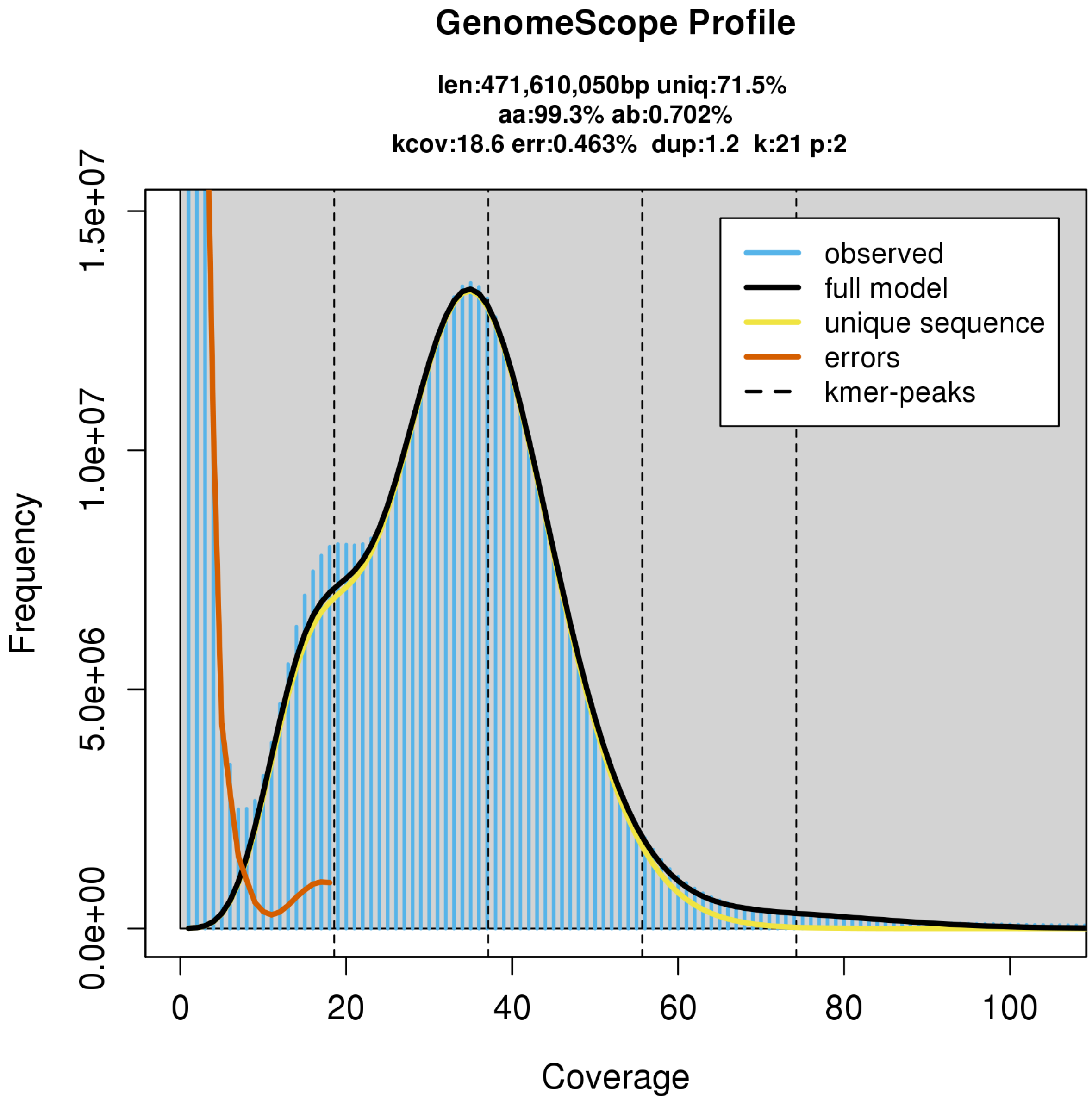

Figure S1: Kmer spectrum and Genomescope2.0 estmates of genome size and heterozygosity.

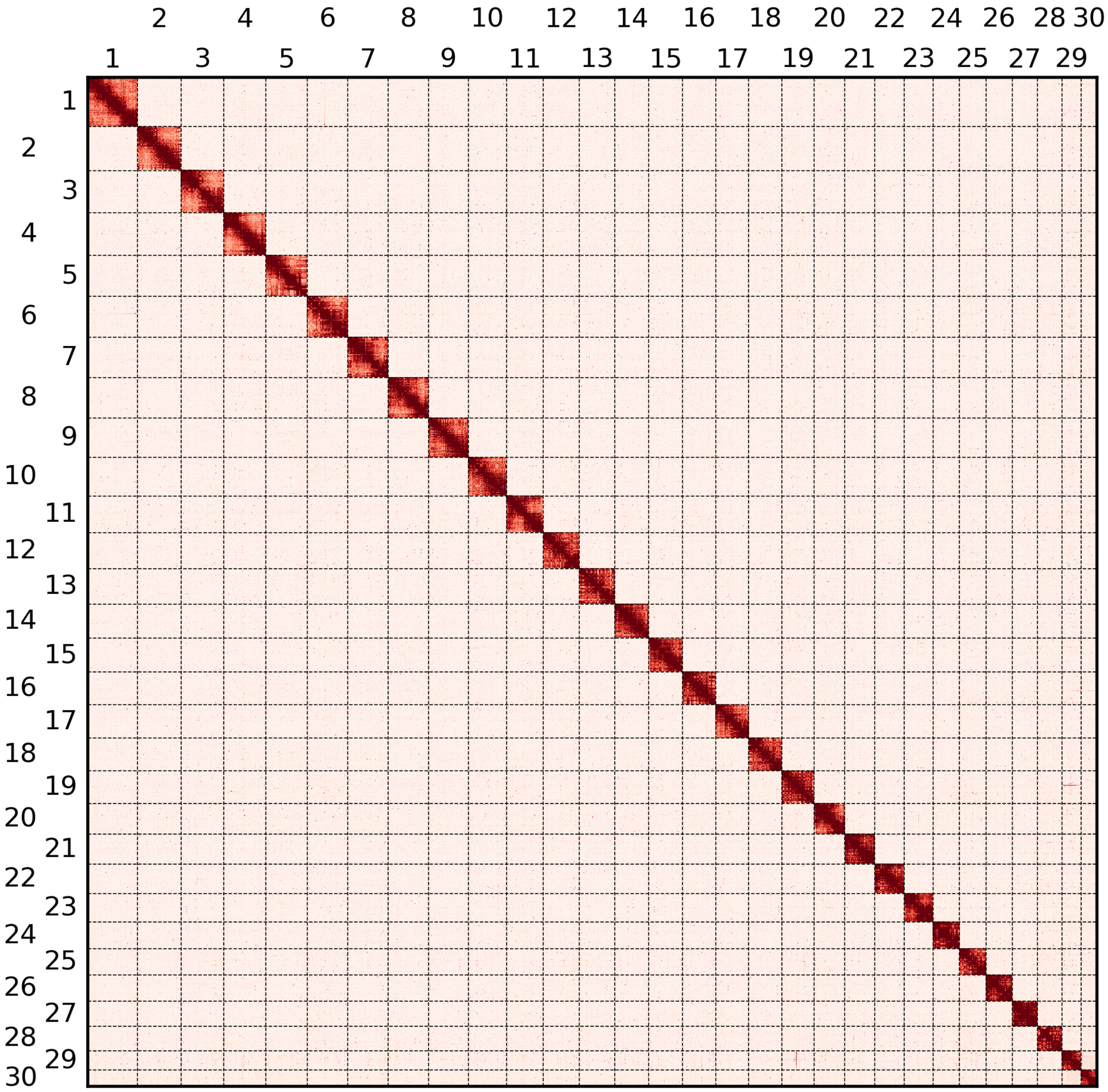

Figure S2: HiC contacts across all 30 *I. podalirius* chromosomes. The intensity of colour is proportional to the number of HiC contacts two regions of the genome share.

Table S1: Annotated transposable elements

Repeat class	No. elements	Total length (Mb)	Percentage of genome (%)	No. distinct classifications
**Retroelement**	244343	86.73	20.15	1152
SINE	120173	28.19	6.55	68
LINE	105889	47.40	11.01	739
Penelope	6955	2.03	0.47	16
LTR element	11326	9.11	2.12	329
**DNA transposon**	39867	13.34	3.10	512
**Rolling Circle**	81955	19.33	4.49	166
**Unclassified**	61052	21.82	5.07	355
**Other**	14	0.01	0.00	2
**Total**	427241	141.23	32.81	2187

Table S2: Chromosome statistics. Note that chromosome 1 is the putative Z chromosome given orthology to other lepidopteran Z chromosomes

**Chromosome**	**Length (Mb)**	**No. gaps**	**Exonic prop.**	**TE prop.**	H_{4D}
1	21.05	9	0.046	0.286	0.00328
2	18.58	2	0.049	0.250	0.00571
3	18.05	1	0.040	0.266	0.00608
4	17.99	3	0.053	0.252	0.00502
5	17.43	13	0.054	0.259	0.00588
6	17.34	5	0.036	0.273	0.00770
7	17.20	5	0.057	0.244	0.00570
8	17.16	8	0.042	0.259	0.00728
9	16.76	4	0.046	0.260	0.00628
10	16.36	5	0.045	0.262	0.00642
11	15.53	5	0.045	0.263	0.00501
12	15.35	4	0.043	0.250	0.00726
13	15.09	4	0.039	0.258	0.00912
14	14.38	5	0.050	0.266	0.00647
15	14.30	8	0.046	0.248	0.00640
16	14.09	7	0.055	0.269	0.00628
17	14.07	16	0.039	0.264	0.00653
18	13.96	9	0.047	0.265	0.00796
19	13.80	5	0.040	0.285	0.00706
20	13.03	11	0.053	0.283	0.00780
21	12.76	2	0.042	0.248	0.00681
22	12.53	1	0.038	0.264	0.00685
23	12.12	1	0.053	0.262	0.00723
24	11.28	2	0.050	0.293	0.00809
25	11.22	13	0.033	0.313	0.01026
26	11.17	0	0.040	0.282	0.00797
27	10.68	3	0.042	0.264	0.00916
28	10.39	5	0.035	0.310	0.00892
29	8.18	16	0.062	0.394	0.00998
30	6.78	14	0.064	0.385	0.00952
All	428.61	186	0.046	0.271	0.00680
